# Non-Immune Functions of Innate Immunity Acting on Physiological Processes: Insights from *Drosophila*

**DOI:** 10.3390/ijms26031087

**Published:** 2025-01-27

**Authors:** Xiaoyan Li, Xiaomin Wang, Zhen Shang, Shan Yang, Yan Tang, Wenhua Xu

**Affiliations:** Institute of Regenerative Medicine and Laboratory Technology Innovation, Qingdao University, Qingdao 266071, China

**Keywords:** innate immunity, physiology, *Drosophila*, behavior, lipid metabolism

## Abstract

As the first line of host immune defense, innate immunity plays a key role in warding off foreign pathogens and damage. *Drosophila melanogaster*, as a classical model animal for more than 100 years, is an important research model for studying innate immunity. In recent years, scientists have made remarkable progress in the recognition mechanisms of innate immunity, the mechanisms of effector molecules, and the modes of their response at the cellular and tissue levels. However, the interaction between innate immunity and other physiological functions remains relatively novel and has yet to be systematically explored. Here, we first briefly discuss the link between the innate immunity system and physiological regulation, from several representative perspectives such as sleep, insulin, and brain function. Then, using *Drosophila* as a model, we provide an overview of the physiological system and specifically summarize the research on the regulation of physiology by innate immunity, covering sleep, lipid metabolism, development, neurodegenerative diseases, memory, feeding, lifespan, movement, and antioxidation. This review provides valuable perspectives into how innate immunity influences other physiological processes, providing a deeper understanding of the complex roles underlying innate immunity.

## 1. Introduction

Innate immunity is ubiquitous across all species, from primitive multicellular organisms to mammals, and as the first line of defense against invading pathogens, it is essential for all organisms to maintain their integrity [1]. Innate immunity recognizes pathogens through pattern recognition receptors (PRRs), initiates immune responses, and activates various immune effector pathways to remove foreign threats [2]. While spectacular progress has been made in the past 25 years in our understanding of the genetic control of innate immunity, we are still lacking knowledge on how it integrates with other physiological functions in multicellular organisms. Indeed, it is important for the maintenance of homeostasis that innate immunity responds to changes in other systems in a sensitive and adaptive manner. In addition, it has become apparent that activation or deactivation of the innate immune system will cause changes in a range of other different system components and can lead to physiological changes in the body relevant to control of the infection [3]. 

To investigate how innate immunity is integrated with other physiological functions, the simple and easily manipulated model organism *Drosophila melanogaster* provides a number of assets. *Drosophila*, a classical model animal for more than a hundred years, has the advantages of a clear genetic background, a short life cycle, a high reproductive capacity, a small size, and the fact that it can be easily grown in the laboratory [4]. Over time, *Drosophila* has played a crucial role in advancing our understanding of innate immunity, notably through the early genetic discovery of the signaling pathways that regulate antimicrobial peptide (AMP) gene expression [5] and the first demonstration of the involvement of the *Toll* gene in the immune response [6]. Several relatively intact innate immunity pathways have been established in *Drosophila*. (i) The Toll pathway recognizes β-glucan, a cell wall component of fungi, and lysine-type peptidoglycan, a cell wall component of Gram-positive bacteria, then activates the transcription of target genes, including the antifungal peptide drospirenine, to exert immune defense [7]. (ii) The IMD pathway is primarily activated by diaminopimelic acid-type peptidoglycan (DAG-PGN) in the cell wall of Gram-negative bacteria. Activation promotes the production of antimicrobial peptides, such as Diptericin or Cecropin, which are released into the hemolymph and coordinate to control systemic infections [8]. (iii) The c-Jun N-terminal kinase (JNK) signaling pathway is activated downstream of Wengen, the receptor for the TNF-related cytokine, Eiger, in *Drosophila*. This pathway is mainly triggered by oxidative stress and plays a key role in controlling apoptosis induced by inflammation [9]. (iv) The Janus Kinase/Signal Transducer and Activator of Transcription (JAK/STAT) pathway is involved in systemic immune responses to tumors, epidermal trauma, and mechanical stress and also has effects on intestinal microbial defense [10]. (v) The Stimulator of Interferon gene (STING) signaling pathway is activated by viral nucleic acids and plays a role in recognizing and defending against viruses [11]. In addition, RNAi, phagocytosis, and melanization also play important roles in the *Drosophila* immune system [12,13]. *Drosophila melanogaster* is a highly complex organism, equipped with numerous sensory organs that detect sound, vision, touch, and so on [14]. These sensory inputs are gathered from the environment and processed by the nervous system, which then translates them into behavioral responses [15]. Numerous behavioral studies have been carried out on *Drosophila*, covering sleep [16], copulation [17], and learning [18]. These investigations range from simple behaviors, like preferences for certain smells and tastes [19], to more intricate behaviors, such as collective group actions [20]. 

More than 65% of human disease-associated genes have homologous genes in *Drosophila*, highlighting the relevance of the fly model for dissecting the genetic regulation of important pathways in humans [21]. In this review, we focus on *Drosophila* as a model to explore the link between innate immunity and physiological processes. These studies remind us that, in humans, when applying innate immunity drugs to treat diseases, it is important to pay attention to the impact on physiological regulation and to prevent potential serious side effects early on. 

## 2. Innate Immunity

Innate immunity, as the primary line of defense, is a critical defense mechanism for all organisms, and it is able to rapidly recognize and initiate a series of signaling responses within hours of pathogen invasion. Innate immunity senses microbial invasion and cellular damage by recognizing pathogen-associated molecular patterns (PAMPs) and damage-associated molecular patterns (DAMPs) through a variety of pattern recognition receptors (PRRs), including Toll-like receptors (TLRs), Nucleotide oligomerization domain-LRR containing receptors (NLRs), RIG–I-like receptors (RLRs), C-type lectin receptors (CLRs), and cGAS-like receptors [22,23]. TLRs are transmembrane receptors containing leucine-rich repeat sequences in their ectodomains, which mediate recognition [24]. NLRs target bacteria and viruses that colonize the cytoplasm, recognizing invasive components within the cytoplasm [25]. RLRs detect viral infections by detecting double-stranded RNAs that are produced during viral replication [26]. CLRs recognize the glycan portion of bacteria and fungi and can also sense molecules associated with dead cells [27]. cGAS is a cytoplasmic receptor that recognizes nucleic acids [28]. Recognition induces the production of a variety of antimicrobial molecules, cytokines, and chemokines in the infected tissues. Investigating the interactions between the immune system and other physiological processes can shed light on how the immune system is linked to different aspects of life. Here, we summarize several advances in currently available experimental studies. 

Sleep is classified as rapid-eye-movement (REM) and non-REM (NREM) and is mainly regulated by self-regulation and the circadian rhythm system [29]. When environmental stimuli, bacteria, and infectious agents are recognized by PRRs, then PRRs produce cytokines like interleukin (IL)-1 and tumor necrosis factor (TNF). Administration of IL-1 suppressed the duration of REM sleep and promoted the duration and intensity of NREM sleep [30,31,32]. When the body receives a stimulus or infection, substances such as TNF and IL-1 reach the brain and promote NREM sleep. However, during severe infections, TNF and IL-1 interfere with both NREM and REM sleep, leading to sleep fragmentation [33]. Although numerous experiments have mutually verified the effects of TNF and IL-1 on sleep, the mechanisms have still not been clearly elaborated [33].

Much attention has also been given to the interaction between insulin and immune inflammation [34]. In obese mice, free fatty acid exposure promoted the infiltration of a variety of immune cells into the adipose tissue of mice, and inflammatory mediators secreted by immune cells, such as TNF and IL-1, induced insulin resistance in adipocytes [35,36]. Meanwhile, it was demonstrated that mice lacking IL-1β or its receptor IL-1R1 were protected from diet-induced insulin resistance [37]. Treatment of rodent diabetes models with IL-1 signaling inhibitors or antibodies targeting IL-1β has also been shown to improve insulin sensitivity [38].

Innate immunity is also implicated in the maintenance of brain function, and over-activation of immunity increases the incidence of neurodegenerative diseases, such as Alzheimer’s disease (AD) [39]. AD is associated with dysregulation of β-amyloid precursor protein (βAPP) metabolism, which leads to transient overproduction or reduced degradation of β-amyloid (Aβ) [40]. Cytokines such as TNFα, IL-1β, IL-6, and transforming growth factor β (TGF-β) can stimulate the synthesis of βAPP when produced at high concentrations for long periods of time. Also, they can induce γ-secretase enzyme activity via Jun N-terminal kinase (JNK) and p38 mitogen-activated protein kinase (MAPK) pathways, which cleaves APP and initiates Aβ formation [41]. Since the 1980s, there have been evident signs that age-related alterations in the innate immune system could play a role in the onset of neurodegenerative disorders like Alzheimer’s disease, and various hypotheses have been established [42,43]. Unfortunately, there is still a large gap in research on immune regulation of neurological disorders until today. 

### 2.1. Physiology of Drosophila

The *Drosophila* nervous system, metabolic pathways, cellular signaling, gene expression regulation, and developmental mechanisms are highly conserved with mammals. Several sensory systems in *Drosophila*, including auditory perception, cognition, and recognition, share structural and functional similarities with those in mammals, including humans. These systems utilize similar or even identical cellular and molecular mechanisms for sensory perception and signal transduction. These conserved features support *Drosophila* as an ideal model for studying physiological processes, disease mechanisms, and gene function [44]. Flies are able to perceive changes in their environment through a multisensory system of sight, smell, touch, and chemoreceptors, which is the basis for complex behaviors [45]. Flies can perceive minute amounts of smell molecules and regulate foraging and mating behavior through chemical signals and pheromones [46]. *Drosophila melanogaster* displays many elaborate and complex behaviors, including, but not limited to, learning, memory, and sleep. Flies have the ability to learn and remember, and they exhibit basic characteristics similar to those of mammals [47]. They are able to learn to run away from specific smell that they have been shocked with or fly toward a smell associated with sugar rewards [48].

The sleep of flies is defined by the alternation of its active and quiescent states [49]. *Drosophila* is considered to be in a sleep state when its behavioral activity is significantly reduced and when it appears to be in a state of quiescence for more than 5 consecutive minutes. The sleep of flies is periodic, usually with two peaks in a day, and shows a 24 h cycle [50]. The sleep cycle of flies is related to the environmental photoperiod, but it also has a certain degree of autonomic rhythm. The sleep cycle is usually 20–30 min and has significant individual differences [16,51]. In addition, *Drosophila* has complex group behavior and has become a well-established model for study [52]. Evidence suggests that group living may facilitate social learning, influence foraging decisions, and enhance sensitivity to environmental stressors [53].

### 2.2. Innate Immunity Acts on Physiology in Drosophila

*Drosophila* relies on multiple layers of defense to ward off invading pathogens. First, it has a hard outer cuticle that provides a physical barrier to keep out bacteria and other pathogens [54]. In addition, the immune system is divided into cellular response and humoral immunity. The cellular immune response occurs rapidly after infection, with macrophage-like blood cells phagocytizing pathogens and crystal cells in larvae inducing melanotic production to promote wound healing [55]. Humoral immunity is triggered within an hour after infection with the release of anti-microbial peptides (AMPs) from the fat body and blood cells to protect the body against foreign invasion [56]. This process involves a variety of signaling pathways, including the Toll, IMD, JNK, and JAK/STAT signaling pathways and their regulatory networks [57,58,59]. In addition, insects also use RNA interference (RNAi), STING-dependent induced responses, and autophagy as mechanisms to fight against viral infection [60]. *Drosophila*, as a well-established research model, not only plays an important role in studies of physiological regulation but also provides unique advantages in studying innate immunity. In recent decades, numerous studies have progressively revealed how innate immunity regulates physiological processes, providing valuable clues to our in-depth understanding of the relationship between the two. 

### 2.3. Sleep and Innate Immunity

Many experimental phenomena suggest a close relationship between sleep and the immune system, which has been especially validated in the *Drosophila* model. Following a bacterial infection, flies exhibit a transient acute increase in sleep [61]. And as the infection persists and leads to exacerbation of the disease, sleep duration gradually decreases and the quality of sleep is significantly reduced [62]. Interestingly, when *Relish*, the immune signaling transcription factor, was mutated, the amount of sleep was significantly reduced compared to normal flies, and the infection-induced sleep changes were not seen in the mutant flies, suggesting that infection-induced sleep changes may be regulated by immune system activation [61]. Further studies have found that by modulating the excitability of neurons in specific regions of brain, enhanced sleep can increase the resistance to infection, enhance the efficiency of clearing bacteria, and ultimately improve the survival rate after infection [63]. Other studies verified the relationship between sleep and immunity from another angle through sleep deprivation experiments. Using mechanical or genetic methods to keep flies awake for a limited period of time, the researchers found that sleep deprivation triggered a series of changes in gene expression, the most significant of which was an increase in the expression of immune-related genes [61]. Enhanced expression of immune genes during sleep deprivation helped to protect flies from bacterial infection. Particularly in the case of mechanical sleep deprivation, post-infection survival was significantly enhanced in a manner associated with restorative sleep [64]. 

Overexpression of *nemuri*, an antimicrobial peptide, significantly increased sleep and arousal thresholds in *Drosophila*, and surprisingly, mutation of *nemuri* had no significant effect on sleep duration but decreased arousal thresholds, suggesting poorer quality sleep [65]. After bacterial infection, overexpression of *nemuri* in the brain promoted increased sleep and improved survival compared to controls. Anatomical analysis revealed that nemuri-expressing cells in the brain project to the fan-shaped body, key neurons that control sleep [65]. In addition, transforming growth factor-beta-activating kinase 1 (TAK1) targets c-Jun N-terminal kinase (JNK) by acting in coordination with an IMD-dependent signaling pathway. Deletion of *TAK1* resulted in the significant reduction of sleep in *Drosophila*, and RNAi knockdown of JNK specifically in the brain also reduced sleep [66]. Overexpression of *PGRP-LC*, which recognizes a peptidoglycan from Gram-negative bacteria and initiates the IMD signaling pathway, also led to reduced sleep duration in *Drosophila* [67]. 

Immunity is closely linked to sleep in a bidirectional manner from *Drosophila* to mammals. Activation of the immune system due to infection or trauma leads to alterations in sleep duration and intensity, and increased sleep early in the course of infection is thought to promote host defense. Sleep affects various immune parameters, and sleep deprivation can lead to inflammation. These phenomena support an interactive link between the immune system and the sleep system. 

### 2.4. Lipid Metabolism and Innate Immunity

In *Drosophila*, the fat body is widely distributed throughout the whole fly and is a major site of immune signaling, performing liver, fat, and immune functions [68]. This facilitates our use of *Drosophila* as a model to elucidate immunometabolic pathways that are highly conserved with mammals. The JNK pathway regulates insulin production and has been implicated in *Drosophila* diabetes. JNK antagonizes Insulin/IGF signaling (IIS) through activation of Forkhead Box O transcription factor (Foxo) and downregulation of *Drosophila* insulin-like peptide 2 (Dilp2) expression [69]. Eiger controls somatotype during substrate deprivation through activation of its receptor, Grindelwald, which acts remotely on insulin-producing cells (IPC) to control body size during substrate deprivation. In the presence of a high-sugar diet, Eiger in adipose bodies prevents hyperglycemia [70]. Activation of the Toll pathway results in decreased triglyceride stores. Expression of *Toll* or its downstream *dMyD88*-induced Toll pathway decreased dAkt phosphorylation in adult female abdominal fat bodies. Triglyceride levels were reduced in fat bodies at 10 h after pathogen infection, and the reduction was suppressed in dMyD88 loss-of-function mutant purist flies [71]. 

STING-deficient flies had reduced lipid stores and downregulated lipid metabolism gene expression, resulting in significant reductions in major storage metabolites such as TAG, alginate, and glycogen [72]. STING has been reported to interact with the lipid synthases acetyl coenzyme a carboxylase (ACC) and fatty acid synthase (FASN). In the fat body, STING is co-localized with ACC and FASN in the cortical region of the endoplasmic reticulum. In in vitro experiments, STING deficiency led to disturbed localization of ACC in fat body cells and a significant decrease in FASN activity [72]. Mutations in another antiviral gene, *Nazo*, led to increased lipid degradation, decreased lipid droplets, and a significant reduction in triglyceride levels in vivo. *Nazo* deletion reduced Perilipin-2, which inhibited the activity of the lipase Brummer, ultimately leading to abnormal lipid metabolism [73]. Excitingly, recent studies have revealed that *Nazo* is a downstream antiviral gene of the STING signaling pathway regulated by the transcription factor Relish [74]. In terms of effect on lipid metabolism, there is also a high degree of congruence between Nazo and STING. Although the available evidence speculates that Nazo regulates lipid degradation while STING acts on lipid synthesis, the link between the two in lipid metabolism remains intriguing [74]. 

There are evolutionarily conserved interactions between the immune response and metabolic regulation, and the proper functions of both are highly integrated and interdependent. Substantial immune receptor activation and intracellular immune signaling are observed in obesity and diabetes. Activation of immune pathways following pathogen infection leads to reduced triglyceride levels in the fat body. In *Drosophila*, the Toll, JNK, and STING pathways are involved in lipid metabolism to varying degrees and have been associated with a variety of diseases, including diabetes and insulin resistance. This suggests that there is a wide range of interactions between the immune system and lipid metabolism, and more mechanisms remain to be explored. 

### 2.5. Development and Innate Immunity

In *Drosophila*, the immune function of plasmocytes is to phagocytose small pathogens, and they are considered equivalent to vertebrate macrophages. After phagocytosis, pathogens are degraded within phagosomes after fusion with endosomes [75]. Phagocytosis plays a key role in nervous system development. During nervous system development, neuroglia regulate cell number and sculpt neural circuits through phagocytosis, including removal of excess apoptotic neurons and pruning of neuronal branches [76]. Matrix metalloproteinase-1 (MMP-1) plays an important role in the neuroglial response to severed axons in *Drosophila* [77]. Notably, glial induction of MMP-1 requires the participation of the highly conserved phagocytic receptor Draper, as well as the transcription factors AP-1 and STAT 92E. In MMP-1-depleted flies, glial cells fail to appropriately clear severed axon regions, leading to a failure to remove degenerating axon fragments and ultimately to the onset of nerve damage [78]. 

In addition, expression of Toll in larval fat bodies resulted in an overall reduction in body size, a developmental delay of approximately 24–36 h, and reduced survival [71]. There is evidence that the Toll pathway may inhibit larval development by interfering with energy supply through inhibition of insulin signaling activity [71]. The Toll pathway is essential for the formation of dorsoventral morphology during embryonic development and is intimately involved in the development of the nervous system [79]. The JAK/STAT pathway plays a crucial role in embryonic development, and its activation stimulates the proliferation of intestinal stem cells (ISCs), enabling their mitotic division in the intestine. Consequently, this pathway is activated by local immune responses in the gut, driving epithelial repair [80]. 

### 2.6. Neurodegenerative Diseases and Innate Immunity

Neurodegenerative diseases are diseases that progressively damage and destroy parts of the nervous system, and common types of diseases include Alzheimer’s disease (AD), Parkinson’s disease (PD), amyotrophic lateral sclerosis (ALS), and frontotemporal dementia (FTD) [81]. The pathological mechanisms underlying AD are associated with abnormalities in amyloid beta peptide (Aβ) and tau. By comparing *w^1118^* control flies with transgenic flies expressing Aβ42 and tau proteins independently, it was found that tau and Aβ42 significantly increased the expression of several antimicrobial peptide (AMP) genes, such as *Attacin A* (*AttA*) and *Diptericin B* (*DptB*), in the aged *Drosophila* brain [82]. Transcriptome analysis showed that the mRNA levels of AMP genes in wild-type flies gradually increased with age, while the AD group showed first a decline and then an increase, suggesting a close interrelationship between AD and AMP genes [83].

In a *Drosophila* model of Parkinson’s disease, *pink1* loss-of-function mutation caused mitochondrial damage that triggered a Relish-mediated immune signaling pathway that expressed antimicrobial peptides, which led to gut dysfunction and neurotoxicity. Inhibition of *Relish* in *pink1* mutant flies restored mitochondrial health and provided neuroprotection [84]. In addition, increased AMP expression in dopaminergic (DA) neurons accelerated neuronal degeneration and increased the likelihood of developing Parkinson’s disease, which could be reversed by knockdown of *Relish* [85]. Indeed, a recent report examined the potential role of STING in Parkinson’s disease by linking STING signaling to the phenotype of flies with early-onset mutations in Parkinson’s disease [86]. This study showed that deletion of *dSting* rescued thoracic muscle atrophy and reduced climbing ability, which was caused by the loss of function of *parkin*. In *parkin* mutants, *dSting* deletion also prevented changes in mitochondrial morphology, suggesting a feedback role for STING in maintaining normal mitochondrial function. Additionally, simultaneous deficiency of parkin and STING inhibited cell death pathways [86]. 

In a *Drosophila* model of ALS/FTD involving neuron-specific expression of poly(GR), RNA-seq analysis revealed that the expression of several antimicrobial peptide genes (AMPs), including *metchnikowin* (*Mtk*) and heat shock protein (*Hsp*), was upregulated, and that this upregulation was regulated by topoisomerase II (TopoII) [87]. Both Mtk knockdown in neurons and Hsp 90 knockdown were able to inhibit retinal degeneration induced by poly(GR) neuronal expression and neurodegeneration in motor neurons. TopoII knockdown also inhibited poly(GR) toxicity in flies, further confirming this story [88]. In addition, in a model where pan-neuronal expression of mutant SOD1 in *Drosophila* led to motor neuron degeneration, transcriptome analysis also revealed upregulation of several AMPs, including Mtk [89]. 

### 2.7. Other Physiological Regulation

**Figure 1:**
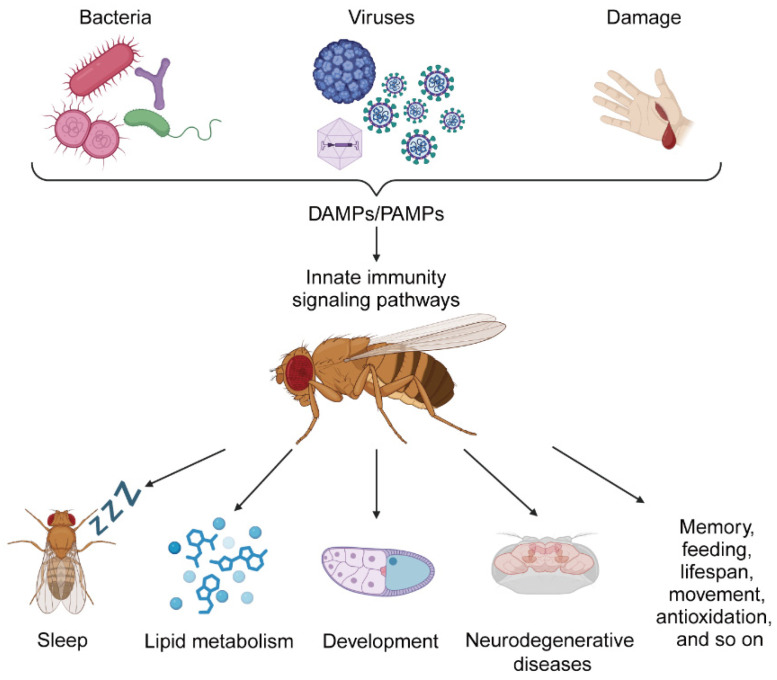
The role of innate immunity in regulating physiology in *Drosophila*.

In addition to the above aspects, many experimental phenomena linking immunity to physiological homeostasis have been observed in a variety of areas involving behavior, antioxidation, lifespan, and more (Figure 1). JAK/STAT signaling was activated in specific regions of the intestine by neighboring male gonads to induce sex differences in intestinal carbohydrate metabolism, which in turn controlled behavioral activities such as food intake and sperm production via gut-derived citrate [90]. PGRP-LC plays a crucial role in initiating and maintaining homeostatic synaptic plasticity. This receptor regulated homeostatic control of the easy-release pool of synaptic vesicles after the inhibition of postsynaptic glutamate receptor activity [91]. Upregulation of PGRP-LC expression resulted in decreased memory capacity [67]. 

Pathogens, such as bacteria and viruses, and damage, are recognized by the immune system as pathogen-associated molecular patterns (PAMPs) and damage-associated molecular patterns (DAMPs) that activate the pattern recognition receptors (PRRs) of innate immunity. In *Drosophila*, PRRs initiate innate immunity signaling pathways and activate a variety of immune effector molecules to remove foreign threats and repair damage. Activated innate immunity pathways also affect physiological regulation in *Drosophila*, such as altering sleep rhythms, affecting lipid metabolism, disrupting development and even causing neurodegenerative diseases. In addition, the innate immunity pathway also regulates memory, feeding, lifespan, movement, antioxidation, and so on. The image was created using BioRender (https://biorender.com) accessed on 24 December 2024.

JNK facilitates the nuclear translocation of Foxo, triggering the Foxo-dependent activation of genes involved in cell-autonomous stress response and repair, which are crucial for cell removal during stress and senescence. Flies in which JNK was mildly activated had longer lifespans than the homozygous wild-type controls, with a significant amplification of DNA damage and reduction of cell cycle arrest during senescence [92]. Overexpression of *bsk* in JNK signaling promoted paraquat resistance in *Drosophila*. Similarly, reducing the gene dose of puc to boost JNK signaling significantly impacted resistance to paraquat, indicating that JNK signaling improves oxidative stress tolerance in *Drosophila*. Notably, the JNK pathway also mediates mitochondrial translocation in response to excess ROS, highlighting its potential importance in neurodegenerative diseases [9,93].

## 3. Conclusions and Discussion

Innate immunity is a natural immune defense system that has gradually developed in organisms during long-term germline evolution [94]. The immune defenses of *Drosophila melanogaster* rely only on innate immunity, as insects lack an adaptive immune system, which makes it a model organism to study innate immunity [95]. This review discusses the non-immune functions of the *Drosophila* innate immunity pathways across a wide range of physiological processes, such as sleep, lipid metabolism, development, neurodegenerative diseases, memory, feeding, lifespan, movement, and antioxidation (Table 1). While the innate immune pathways are stimulated to perform anti-infective and anti-tumor functions, their activation also affects behavior, metabolism, and so on. In many cases, the absence of immune molecules not only leads to abnormalities in immune responses but also interferes with the maintenance of homeostasis of non-immune physiological systems. 

Sleep has been shown to be closely related to immunity in both mammals and *Drosophila* [33,65]. Activation of the immune system caused by infection or trauma can interfere with normal sleep, and changes in sleep can, in turn, affect immune effects [62]. However, in experiments, individual differences in sleep are large and strongly influenced by genetic background, and environmental factors such as temperature and humidity can also interfere with the results, so researchers need to analyze the experimental data more carefully and try to corroborate data from multiple perspectives to ensure the credibility of the results. In *Drosophila*, the Toll, JNK, and STING pathways are all involved in lipid metabolism. A reasonable guess for the existence of a close connection between the immune system and metabolism is that, faced with pathogen invasion, the body needs to raise a large amount of energy through metabolic pathways to supply the immune system to resist the attack. Meanwhile, conserved inflammatory metabolism has been abundantly reported in mammals, providing a basis from an evolutionary perspective [96,97]. On the one hand, with respect to sleep and lipid metabolism, there is a large body of experimental evidence showing that after infection, the proper physiological processes of sleep and lipid metabolism undergo significant alterations regulated by innate immune pathways. At the same time, abnormal expression of numerous immune genes has been observed in conditions such as sleep deprivation and obesity. These findings support the existence of a functional link between the immune system and both the sleep and lipid metabolism systems, with these systems intertwined in a complex network. On the other hand, for other physiological processes involved, such as development, neurodegenerative diseases, etc., research often focuses solely on demonstrating that a particular innate immune gene has a role in regulating physiological processes, and does not address the interrelationships between the immune and non-immune functions of that gene. For this section, our overview emphasizes the growing evidence and interest in the multiple roles of innate immunity genes in various physiological processes. Further research is necessary to explore whether there are causal, demonstrable, and substantial interactions between these systems and the immune system, beyond the mere sharing of a particular gene.

An overview of the link between innate immunity and physiological regulation contributes to a better understanding of how multiple systems synergize with each other to defend against viral invasion and maintain organismal homeostasis. From a multisystem perspective, following infection, activation of the immune system triggers a series of systemic systemic changes that could lead to abnormal physiological and behavioral alterations [98]. These changes may provide an additional protective effect to the organism in some cases, but they may also result in complications. Despite many important advances in recent years, most of the links between innate immunity and many physiological processes have been phenotypic in nature and have not been further explored at the molecular level. The fact that these mechanisms are often complex and intertwined has resulted in existing studies often remaining fragmented. Extrapolated from *Drosophila* to *mammals* and *humans*, it reminds us that the application of innate immunity requires that there be no serious disruptions to the various physiological systems. The translation of experimental findings into effective clinical interventions requires the consideration of multisystem effects.

## Figures and Tables

**Table 1:** Innate immunity-related genes involved in physiological regulation in *Drosophila*.

Genes	Physiological Processes Involved	References
*Toll*	Triglyceride stores	[71]
	Development of the nervous system and dorsoventral morphology	[79]
*Relish*	Sleep	[61,62,63,64]
	Parkinson’s disease	[84,85]
*JNK*	Insulin production and diabetes	[69]
	Sleep	[66]
	Lifespan	[92]
	Paraquat resistance and oxidative stress tolerance	[9,93]
*JAK/STAT*	Proliferation of intestinal stem cells (ISCs)	[80]
	Intestinal carbohydrate metabolism	[90]
	Food intake	[90]
	Sperm production	[90]
*Sting*	Lipid metabolism	[72]
	Parkinson’s disease	[86]
*Nazo*	Lipid droplet and triglyceride levels	[73]
*PGRP-LC*	Sleep	[67]
	Synaptic plasticity	[91]
	Memory	[67]
*TAK1*	Sleep	[66]
*nemuri*	Sleep quality and arousal thresholds	[65]
*Attacin A*	Alzheimer’s disease	[82,83]
*Diptericin B*	Alzheimer’s disease	[82,83]
*metchnikowin*	Amyotrophic lateral sclerosis (ALS) and frontotemporal dementia (FTD)	[88,89]
*Hsp*	Amyotrophic lateral sclerosis (ALS) and frontotemporal dementia (FTD)	[88]
